# Retrospective review of the epidemiology, microbiology, management and outcomes of intra-cranial abscesses at a neurosurgical tertiary referral centre, 2018–2020

**DOI:** 10.1186/s12941-022-00550-2

**Published:** 2022-12-27

**Authors:** Terry John Evans, Sarah Jawad, Nida Kalyal, Angelina Nadarajah, Meriem Amarouche, Simon Stapleton, Christopher Ward, Aodhan Breathnach

**Affiliations:** St George’s University Hospitals NHS Foundation Trust, London, UK

**Keywords:** Brain abscesses, Intracranial infection, Neurosurgery, OPAT, Oral switch

## Abstract

**Background:**

Intracranial abscesses are rare but serious, and are associated with significant morbidity and mortality. Due to both the rarity and severity of these infections, well-controlled trials have not been reported in the literature, and optimal management is a matter for expert opinion. Advances in surgical management have improved outcomes and increased rates of microbiological diagnosis. However, the approach to antimicrobial chemotherapy varies considerably, including the choice of antibiotic, the duration of treatment, and the timing of oral switch.

**Methods:**

We conducted a retrospective review of 43 cases of intracranial abscesses from a large, tertiary neurosurgical centre in London, UK, between 2018 and 2020, including 29 primary intra-parenchymal abscesses, 11 subdural abscesses and 3 extradural abscesses.

**Results:**

The majority of cases had surgical intervention; 6/43 (14%) required repeat intervention (all intra-parenchymal abscesses). A microbiological diagnosis was made in 83% of cases. Intravenous antibiotics were given for a median of 33 days (IQR 23–44 days), with a variable duration of oral follow-on antibiotics. Total duration of antibiotic treatment ranged from 0 to 467 days. Only three patients from our cohort are known to have died.

**Conclusion:**

Shorter courses of intravenous antibiotics for brain abscesses were not associated with increased mortality. In the absence of well-controlled trials, a national registry of intracranial abscesses would provide invaluable data to inform optimal treatment.

**Supplementary Information:**

The online version contains supplementary material available at 10.1186/s12941-022-00550-2.

## Background

Intracranial abscesses include primary parenchymal abscesses, subdural empyemas and extradural empyemas. Parenchymal abscesses are found within the brain tissue proper. In contrast, subdural and extradural empyemas are found outside the brain but within the skull, and are differentiated based on their relation to the dura mater. They were previously associated with a mortality of up to 40%, and although in recent years this has improved, morbidity can remain significant [1]. These infections remain rare, and may arise following local invasion (for example, in the setting of otitis externa or sinusitis), following haematological spread (as seen in endocarditis), or following trauma. All three entities may occur as post-surgical complications. Spontaneous intracranial abscesses have several associated risk factors, including immunosuppression, intravenous drug use, cardiac abnormalities such as congenital structural defects and infective endocarditis, and anatomically adjacent foci of infection such as in the sinuses, middle or inner ear and dental abscesses. Ideally, treatment of abscesses involves surgical drainage of the abscess, followed by a prolonged course of antibiotics. However, carefully controlled trials have not been reported in the literature, and as a result, management of these complex infections is based on retrospective case series and expert opinion [2].

The duration of intravenous treatment for a number of deep-seated infections is highly topical, and has been the subject of a number of recent landmark trials. Shorter intravenous courses and an early oral switch have been shown to be non-inferior in a variety of infections. For example, the OVIVA trial [3] was a multi-centre randomised controlled trial for bone and joint infections, and the POET study investigated the treatment of infective endocarditis [4]. Based on these studies, early oral switch in these infections has become the standard of care in many centres, particularly when there has been good surgical source control, accompanied by good clinical and biochemical response. However, no such trial for brain abscesses has been conducted.

In 2000, a working party of the British Society for Antimicrobial Chemotherapy published treatment guidelines for brain abscesses, but these were not prescriptive and allowed a wide range of practice [5]. Case series have continued to appear in the literature, and gradual shifts in the microbiological causes and treatments have been seen over time. For example, the predominance of *S. aureus* has now been replaced with the *S. milleri* group (which includes *S. constellatus, S. anginosus* and *S. intermedius*) as the commonest species identified in the UK [6, 7]. The duration of intravenous antibiotics was two or three weeks when described in the earlier literature [8, 9]. In contrast, a recent series from Oxford described a uniform approach to most cases, with the majority of patients receiving 6 weeks’ intravenous antibiotics (usually ceftriaxone) before a further oral course of variable length [6].

An Italian consensus statement [10] stated that when treated surgically, a total antibiotic duration of 4 to 6 weeks is sufficient. Brouwer et al. [1] stated that prolonged intravenous courses are typically required (6–8 weeks), although they concede that 1–2 weeks of intravenous antibiotics may be appropriate before an oral switch, dependent on clinical progress. Thus, a re-evaluation of current approaches is required, in order to provide a more robust evidence base for antibiotic treatment of brain abscesses.

St George’s Hospital, London, is a tertiary neurosurgical referral hospital in south London serving a large geographical area. For this series, we collected clinical, radiological and laboratory data to ascertain trends in the management of brain abscesses and clinical outcomes. We specifically looked at organisms identified; length of intravenous antimicrobial courses and whether shorter courses were sufficient for optimal outcome; surgical management; and morbidity and mortality of cases. In particular we assessed whether our hospital’s guideline recommending two weeks of intravenous antibiotics (see Additional file 1 for our guidelines) was being adhered to, and the clinical outcomes when shorter durations of intravenous antibiotics were used.

## Methods

### Case finding

All primary/spontaneous abscesses treated at our hospital between 2018 and 2020 were included, including all intracranial abscesses, and subdural and extradural collections. Surgical site infections were excluded (defined as occurring within 3 months of surgery), as were infections following trauma. Paediatric cases were included, but neonatal (defined as under 6 months of age) ones were not. Cases were identified using the following ICD-10 codes: G06 (intracranial abscess and granuloma), G061 (intraspinal abscess and granuloma), G062 (extradural and subdural abscess, unspecified), and G07X (intracranial and intraspinal abscess and granuloma in diseases classified elsewhere). Medical records of all cases identified by clinical coding were reviewed to confirm they met the case definition. All data was anonymised prior to analysis. This audit was registered according to local protocols in our hospital.

### Data extraction

All data was obtained from electronic patient records. Where patients had been repatriated to local hospitals, reasonable efforts were made to obtain letters from those institutions for follow-up information, but this was unobtainable in a number of cases. No further data was collected after 30th December 2020.

### Microbiology

Microbiological samples were processed in our UKAS-accredited laboratory, with methods based on national Standards for Microbiological Investigations published by Public Health England. Blood cultures were inoculated into BACTEC bottles and incubated on the Becton Dickinson BACTEC FX system. Pus from brain abscesses was taken urgently to the laboratory and a Gram stain performed. The sample was then inoculated onto blood, chocolate and MacConkey agar incubated in an atmosphere supplemented with 5% carbon dioxide at 35–37 ℃ , and blood agar supplemented with neomycin in anaerobic conditions, with metronidazole discs, at 35–37 ℃. Organisms were identified by mass spectrometry via MALDI-TOF (matrix-assisted laser desorption/ionisation-time of flight). Antibiotic susceptibility was determined using the automated Phoenix platform, and/or antibiotic disc diffusion testing as per EUCAST [11]. In the case of streptococci, the minimum inhibitory concentration (MIC) was measured to determine penicillin susceptibility; breakpoints were defined as per the European Committee on Antimicrobial Susceptibility Testing (EUCAST).

Where surgery was not performed, or intra-operative samples were not available, other microbiological samples yielding the likely pathogen were considered – including blood cultures, and ear swabs for otogenic sources of abscesses.

16S ribosomal RNA PCR analysis of pus was undertaken at Great Ormond Street Hospital for molecular identification of pathogens when none had grown following conventional culture techniques described above.

## Results

### Patient demographics

Between 2018 and 2020, 43 patients were admitted to our hospital with intracranial infected collections that fitted our inclusion criteria: 29 intraparenchymal abscesses, 11 subdural empyemas, and 3 extradural empyemas. The demographic characteristics are summarised in Table 1. The median age of patients with subdural and extradural empyemas (24 years and 14 years, respectively) was younger than those with parenchymal abscesses (50 years) - reflecting the differing aetiologies of these clinical entities. For all three infections, men were over-represented. 63% of patients were transferred to our tertiary referral centre from other hospitals.

**Table 1:** Baseline demographics, risk factors, and presenting symptoms for cases included in this series

	Intraparenchymal abscess	Subdural empyema	Extradural empyema
Number	29	11	3
Male:Female	20:9	9:2	2:1
Age (IQR)	50 (42–57)	24 (15.5–39)	14 (7.5–24)
Mortality	10.3%	0%	0%
Risk factors			
IVDU^a^	2/29	2/11	0/3
Immunosuppressed	4/29	0/11	0/3
Cardiac anomaly	1/29	0/11	0/3
Diabetes	5/29	0/11	0/3
Source			
Dental	6/29	0/11	0/3
Sinus	2/29	7/11	2/3
Ear	4/29	2/11	1/3
Presenting symptoms			
Headache	15/29	10/11	2/3
Fever	6/29	6/11	1/3
Focal neurology	16/29	3/11	0/3
Seizures	8/29	5/11	1/3

^a^Intravenous drug user

### Surgical management

All patients except five had surgical drainage. Four of the five patients who did not undergo surgery presented with intra-cranial abscesses, and one with a subdural abscess. Specifically, one case had microabscesses not amenable to surgery; one patient’s abscess was adjacent to a functionally important area of brain in the posterior frontal lobe; one was a skin infection that had eroded through the skull; one was a haemato-oncology patient for whom surgery was deemed to pose too great a risk; and one was a shallow abscess adjacent to an area of mastoiditis presumed due to *S. pneumoniae* which had been grown in blood cultures.

Surgical techniques included burr hole aspiration, and access by craniectomy and craniotomy. The AxiEM system was used for radiological guidance in some cases, along with intra-operative ultrasonography to determine abscess size.

Six patients required a further aspiration or drainage, mostly due to increased volume of abscess noted on repeat imaging. Typically these patients had non-specific symptoms, such as headache, which prompted repeat imaging. The repeat aspirations occurred between 4 and 23 days after the first surgery. Pus sampled from repeat procedures was sterile in all cases except one. All patients were still on intravenous antibiotics at the time of repeat surgery, and thus an early switch to oral antibiotics did not contribute to the need for repeat surgery.

### Microbiological diagnosis

The causative pathogens of the infections in this series are described in Table 2. A microbiological diagnosis was obtained for 25/29 (86%) of intraparenchymal infections, and 100% of extradural and subdural collections. Consistent with other recent reports [6], the *S. milleri* complex were the commonest species implicated in intraparenchymal abscesses. Significantly however, a very wide range of species was identified, including Gram-negative organisms, mixed organisms, and rarer species such as *Nocardia* and *Actinomyces*. The sources for the infections with *Actinomyces* and *Nocardia* were not clear, and no evident sinus or dental infections were noted. One patient with *Nocardia* had a CT scan of his chest, abdomen and pelvis, which was unremarkable. The second patient with *Nocardia* was immunosuppressed due to non-Hodgkin lymphoma and the infection had disseminated to his prosthetic knee (which required washing out) and to his lung. The patient with *Actinomyces* infection was reviewed by the maxillofacial surgeons and had an unremarkable orthopantomogram. However, *S. oralis* was also isolated from his abscess, indicating a probable oral source. As a proportion of the total, the *S. milleri* complex was detected in 12/29 (41%) of intraparenchymal brain abscesses. Notably, there were no cases caused by *Staph aureus* (discussed further below).

13 of the intra-operative samples had no organisms seen on Gram staining; this is not uncommon when examining pus, no matter the site of origin. Nonetheless, of these, only two went on to have a negative culture. One of these was subject to 16S rRNA PCR sequencing, and *E. coli* was identified as the causative pathogen.

**Table 2:** Microbiological diagnoses of infections included in this study

	Intraparenchymal abscess	Subdural empyema	Extradural empyema
Gram-positive cocci^a^	0	0	1
*S. milleri*	10	5	0
*S. milleri* mixed^b^	2	2	1
*S. pyogenes*	0	1	0
*S. pneumoniae*	1	1	0
*E. coli*	0	1	0
*Enterobacter cloacae*	1	0	0
*Proteus mirabilis* and anaerobes	1	0	0
*Actinobacillus*	0	0	1
*Actinomyces meyeri*	1	0	0
*Nocardia* sp.	2	0	0
Anaerobes	3	0	0
*Mixed organisms*	4^c^	1^d^	0
Sterile culture	2	0	0
No sample received	2	0	0
TOTAL	29	11	3

The method of identification is described further in the text. Coagulase-negative staphylococci were considered contaminants when grown in mixed populations

^a^Gram positive cocci seen on Gram stain, no organisms grown, not subject to 16S PCR

^b^*S. milleri* grown, with any other organism either grown, seen on Gram stain, or detected by 16S rRNA PCR

^c^Mixtures were: *Actinomyces* sp. and *S. oralis*; *Propionibacterium acnes* grown and Gram-positive rods and cocci seen; *E*. *coli* and *Bacteroides ovatus*; *Staph capitis*, *S. mitis* and *Actinomyces meyeri*

^d^Group C *Streptococcus* and anaerobes

### Antibiotic therapy

**Fig. 1:**
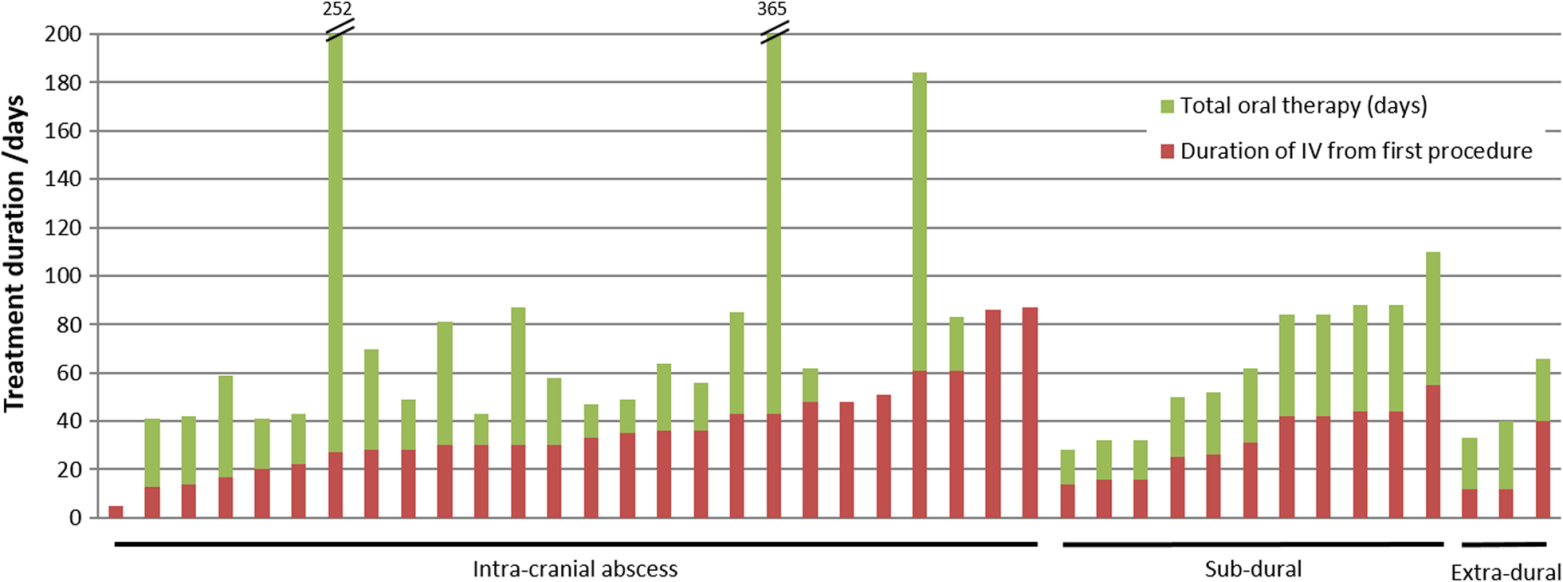
Duration of antibiotic treatment. 3 cases are not represented: 1 case where no antibiotic information is available; and 1 infection each with *Actinomyces* sp. and *Nocardia*, which were treated with prolonged courses of antibiotics (these cases are described further in the text)

The duration of antibiotic treatment for all cases is displayed in Fig. 1. 26/43 (60%) patients who were successfully treated received under 6 weeks’ intravenous therapy (median 32 days, IQR 24–44 days), with five of those patients receiving 2 weeks or less. The shortest duration of intravenous therapy in a surviving patient with an intraparenchymal abscess was 13 days; the causative organism was *S. milleri*. Initial treatment was with ceftriaxone in the majority of cases of intraparenchymal abscesses (22/29). Of the remaining 7 patients with intraparenchymal abscesses, 4 received meropenem initially, one received amikacin and co-amoxiclav (the infection was due to *Nocardia* sp.), one received amikacin and vancomycin (due to a history of anaphylaxis to penicillin), and the final one received initial therapy with benzylpenicillin. In five cases, all in the intraparenchymal abscesses cohort, the antibiotic spectrum was narrowed from ceftriaxone to a penicillin-based antimicrobial. The most frequently used oral antimicrobial was co-amoxiclav (13/43) which was often boosted in the intra-parenchymal cohort with additional oral amoxicillin (625 mg co-amoxiclav three times a day with 500 mg amoxicillin three times a day). Other combinations included oral amoxicillin (1 g three times a day) with metronidazole (9/43), oral co-trimoxazole (3/43; 2 of whom had *Nocardia* sp. infection and 1 had a documented allergy to penicillin) or oral linezolid (2/43; one of whom had *Nocardia* sp. infection and one had a history of penicillin anaphylaxis and a *S. pneumoniae* infection). For those patients who were not repatriated to local hospitals, oral antibiotic choices were made in conjunction with the OPAT (outpatient parenteral antibiotic therapy) team; 19/43 (44%) were discharged home on intravenous antibiotics with the OPAT service, who continued to follow up and monitor the patient for the duration of their treatment.

One patient with *Nocardia* infection, not shown in Fig. 1, received intravenous therapy for 182 days, followed by oral therapy for 467 days. Treatment was complicated by transaminitis caused by co-trimoxazole, and linezolid-induced bone marrow suppression. He survived with no neurological deficit and was discharged from follow-up one year after completing treatment. The patient with *Actinomyces* was treated with intravenous antibiotics for 39 days, followed by oral therapy for 329 days; he made a full recovery. His treatment was complicated by profound neutropenia associated with ceftriaxone use.

### Other medical treatments

Anti-epileptic drugs (AEDs) were used variably across the cases. 13/43 (30%) patients presented with either focal or generalized seizures. Of those not presenting with seizures, one-third did not receive AED. For patients with intra-parenchymal abscesses, primary prophylaxis was prescribed for 14 patients (48%), and secondary prophylaxis was prescribed for 8 patients (28%). For subdural abscesses, primary prophylaxis was give to 6 patients (55%) and secondary prophylaxis was given to 5 (45%); for extradural abscesses, 1 patient each received primary and secondary prophylaxis. The duration of AED treatment is unknown in some cases due to repatriation of patients to local hospitals (11 patients with intra-parenchymal abscesses and 4 with subdural abscesses were repatriated), but 17/43 patients were still taking AEDs at the time of the last follow-up.

59% (17/29) of patients presenting with intra-parenchymal abscesses were given corticosteroids on admission, with a duration of up to 51 days (median 17.5 days, IQR 9.5–25). The factors leading to administration of corticosteroids in individual cases were not always clear.

### Mortality rate

Only three patients in this cohort for whom follow-up data is complete have died. All three had an intraparenchymal abscess (3/29; 10%). One of the patients who died did so after repatriation to his local hospital; he was receiving intravenous antibiotics at the time of repatriation. A second patient had disseminated *Nocardia* infection on a background of non-Hodgkin lymphoma; the death was not felt to be related to the brain abscesses and this patient had received 28 days’ intravenous antibiotics.

The other death occurred in a patient who received 22 days of intravenous antibiotics before a further 21 days of oral antibiotics. Although the brain abscess is thought to have contributed to death, the patient also had cancer and had had recent chemotherapy.

### Discharge and readmissions

15/43 patients were repatriated to their local hospitals and comprehensive follow-up data was often difficult to obtain. A further 19 patients were discharged on intravenous antibiotics under the care of the OPAT team—see Table 3. The length of stay in our hospital was significantly longer for those who completed their intravenous antibiotics in our hospital (median 44 days, IQR 22–70 days) compared to those who were discharged on intravenous antibiotics (median 16.5 days, IQR 13–31 days). However, this difference is likely to be largely explained by the difference in severity of the patients’ clinical condition between the two groups.

**Table 3:** Discharge destinations of patients according to infection type

	Intraparenchymal abscess	Subdural empyema	Extradural empyema
Home	5	3	0
OPAT	12	4	3
Repatriated	11	4	0
Died as inpatient	1	0	0
Total	29	11	3

For those who completed treatment at our hospital, either as inpatients or as outpatients under the OPAT team, five patients with intraparenchymal abscesses were recorded as being readmitted for varying reasons, most of which were deemed unrelated to their presenting complaint. Two patients in the extradural cohort were readmitted, one due to seizures who was managed medically and one for repeat surgical drainage.

## Discussion

Primary intracranial collections remain rare, with about a dozen being treated at our tertiary centre each year. Untreated infections carry a very high mortality rate, and in the absence of trial data, there has been a trend to prolonging antibiotic durations presumably because the grave risk of under-treatment and risks associated with repeat surgery. It is unclear whether the increased provision of intravenous antibiotic administration at home via OPAT services may have contributed to this.

Our hospital’s protocol for the treatment of brain abscesses follows the tenets of the UK’s Start Smart Then Focus campaign [12]. Our guidelines suggest treating with 2 weeks of intravenous antibiotics followed by an oral switch, assuming satisfactory surgical intervention, and good clinical and biochemical response. Our protocol emphasises choosing antibiotics with as narrow a spectrum as possible, guided by the microbiology results. Nonetheless, this case series demonstrates that a significant proportion of patients do receive prolonged courses of third-generation cephalosporins or even carbapenems. The reasons are not always clear, but likely include clinical and biochemical progress of the patient as well as the experience and judgement of the neurosurgeon and microbiologist involved.

In terms of brain abscesses, negative cultures are reported in up to 30% of samples [13]. Thus, our microbiological diagnosis rate was favourable at 86%. We found a wide range of causative organisms, and this underscores the key role for microbiological diagnosis. Notably, there were no cases caused by *S. aureus*. Because of their strong tendency to disseminate, including to the brain, *S. aureus* bacteraemias are now treated aggressively–often for up to 4 weeks where early source control has not been achieved. This may explain the reduction in the number of brain abscesses caused by *S. aureus*. Whilst an unpublished series from Cambridge found *S. aureus* accounted for 9% of brain abscesses [7], only a single case out of 47 was reported in a recent Oxford cohort [6].

In our previous case series [14], the usual empiric treatment was cefotaxime and metronidazole. There has been a shift in our practice to ceftriaxone as the cephalosporin of choice–in part due to the convenience of once-daily dosing, which enables outpatient administration.

The duration of IV treatment varied significantly in our patients, suggesting that the treatment was adjusted to the individual circumstances of the patient and individual preferences of the clinicians and microbiologists. Planned durations were frequently extended due to temporary worsening of the patients’ symptoms, which in some cases were non-specific, such as headache. It was not usually obvious that these symptoms were a result of uncontrolled infection-and in fact, there was usually biochemical evidence that the infection had responded to treatment. In contrast, the majority of cases described by Darlow et al. [6] were treated with intravenous antibiotics for 6 weeks (with a variable duration of oral antibiotics). Our data suggests that a more individualised approach is possible and is safe for many patients, with shorter intravenous courses and antibiotics with narrower spectra of activity. This is supported by data from a retrospective cohort study conducted in France [15]. Further recent evidence comes from Skoutelis et al. [16] who presented a series of patients with smaller abscesses (< 3 cm), all of whom were successfully treated with under 2 weeks of intravenous antibiotics.

Imaging did sometimes demonstrate apparent re-accumulation of the abscess. Where repeat aspiration of the abscess was performed, this yielded sterile material in all cases except one. Therefore, it is possible that the re-accumulation of fluid is an inflammatory or serous exudate rather than ongoing active infection. This is important because the need for repeat surgical intervention was often a justification to extend the intravenous course of antibiotics. However, we found no data to suggest that the clinical outcome in the cases where repeat aspiration was performed would have been affected by an earlier oral switch.

We agree with Darlow et al. [6] that the route of administration of antibiotics is an important outstanding question in the treatment of brain abscesses. The data presented in this retrospective series shows that, at least in a selected patient population, an early oral switch is safe and effective. The potential benefits of an early oral switch include reduced hospital stay, reduced spending on services such as outpatient intravenous antibiotics, improved patient experience, fewer contacts with healthcare, and reduced need for long lines and their associated risks of infection and thrombus (discussed in [17]).

Follow-up of patients in this recent series is short, particularly for the 2020 cohort. Our mortality rate compares very favourably to our series from the early 2000s (10%) and a case series from Oxford (21%). A recent, unpublished, 5-year case series from Cambridge, UK, reported a 10% mortality [7]. It is unclear if our outcomes are significantly different, although there is no signal in our outcome data to suggest that our approach, including shorter durations of intravenous antibiotic courses, leads to less favourable outcomes.

Management of seizure prophylaxis was heterogenous. This is an important issue because it affects the patient’s ability to drive under UK law (https://www.gov.uk/brain-abscess-cyst-encephalitis-driving), and side effects of anti-epileptic medications include fatigue and mood disturbance. There are no clear guidelines on the use of anti-epileptic medications in this setting, and case series are typically silent on this issue. For secondary prevention, the need for anti-epileptic medication is clear. Whilst seizures are common in these patients, there is less clarity regarding primary prophylaxis in those patients who did not present with seizures.

Steroids work to reduce inflammation and oedema, but evidence for their use in intracranial infections is also lacking. The evidence that does exist focuses on meningitis rather than abscesses. Depending on the causative organism, use of steroid may be beneficial, harmful, or of no consequence—and this has recently been reviewed [18]. Animal studies have shown reduced concentrations of antibiotics in brain tissue when co-administered with steroids [19]. It should not be assumed that this intervention will benefit all patients with intra-cranial abscesses, because steroids impair wound healing, contribute to hyper-glycaemia, and are immunosuppressants.

An additional aspect of management is the frequency of neuroimaging during and after treatment. Some centres recommend fortnightly imaging for all patients (as advocated by Brouwer [1]). The balance has to be struck between monitoring disease progression, and appropriate stewardship of investigations. A pragmatic approach was taken at our hospital, with repeat imaging usually performed at the end of treatment, or if the clinical picture suggested it would be helpful to management.

Our results illustrate the benefit of an individualised approach in such a complex condition, and the role for dedicated microbiologists with a special interest in the field. Unified, national guidance would be welcome for the management of these serious and challenging infections but ought not be too prescriptive or risk-averse so as to limit this freedom. A national registry of brain abscess and other intra-cranial infections would provide invaluable data, and may lead to a ‘paradigm reversion’ to shorter intravenous courses of antibiotics. In particular, our case series, and other recent series, raise outstanding research questions:

- What is the optimal antibiotic given the changing microbiology of brain abscesses?
- What duration of intravenous antibiotics is required when there has been optimal surgical management?
- What is the role of peri-operative steroids?
- When and for how long should antiepileptic medications be used?
- What is the role of routine imaging in the follow-up of these patients?

## Limitations

Our hospital is a tertiary neurosurgical referral centre, and it is standard practice for post-operative patients to be repatriated to their local hospitals when clinically stable. This was the case for 11 patients with intraparenchymal abscesses and 4 patients with subdural abscesses. Follow-up data was not available in most of these cases. All patients were repatriated with an antibiotic plan in place, but it was not always possible to be certain of the treatment the patient finally received. However, we anticipate that if there were significant complications, then the patient would have been discussed with our neurosurgical team.

This was a retrospective cohort study. Treatment choice was affected by clinical progress, and therefore we cannot say whether the outcomes in cases when more than 2 weeks of intravenous antibiotics were given would have been different if the oral switch occurred sooner. A controlled trial is not pragmatic for this condition.

In common with other published case series, this study lacks long-term follow-up data. Whilst mortality is likely to be highest at the time of diagnosis and soon thereafter, long-term morbidity and disability have not been assessed in this study.

## Supplementary Information

**Additional file 1.** Antibiotic guideline for the treatment of intracranial abscesses used in our hospital.

## Data Availability

The raw data is stored on a secure system at St George’s University Hospitals NHS Foundation Trust.
