# Supplementary figures and images for: Retrospective review of the epidemiology, microbiology, management and outcomes of intra-cranial abscesses at a neurosurgical tertiary referral centre, 2018–2020
Source: Ann Clin Microbiol Antimicrob. 2022 Dec 27;21:58. doi: 10.1186/s12941-022-00550-2 (PMC9795649; doi:10.1186/s12941-022-00550-2)

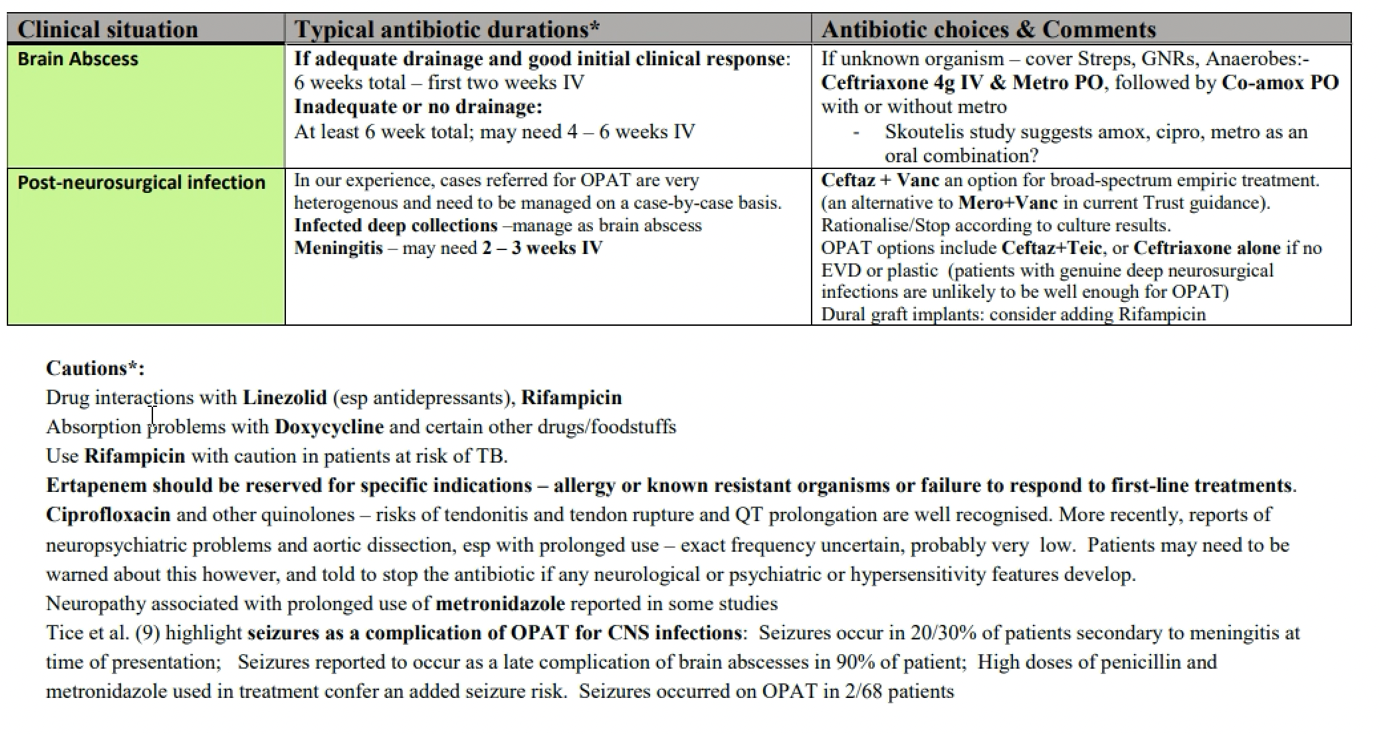

Supplement: Supplementary file 1 — Additional file 1. Antibiotic guideline for the treatment of intracranial abscesses used in our hospital. [file 12941_2022_550_MOESM1_ESM.docx]
